# ChatGPT Dependency Disorder in Healthcare Practice: An Editorial

**DOI:** 10.7759/cureus.66155

**Published:** 2024-08-05

**Authors:** Anamika Chakraborty Samant, Ishaan Tyagi, Jyothi Vybhavi, Hemali Jha, Jitendra Patel

**Affiliations:** 1 Internal Medicine, Chaitanya Hospital and Obesity Centre, Mumbai, IND; 2 Internal Medicine, Dr. D. Y. Patil Medical College, Hospital and Research Centre, Pune, IND; 3 Physiology, RajaRajeswari Medical College and Hospital, Bangalore, IND; 4 Internal Medicine, Integral Institute of Medical Sciences and Research Centre, Lucknow, IND; 5 Physiology, Gujarat Medical Education and Research Society Medical College, Vadnagar, IND

**Keywords:** patient safety, healthcare professionals, clinical workflows, healthcare practice, ai chatbots, chatgpt dependency disorder

## Abstract

Dependency on ChatGPT is characterized by excessive reliance on AI-driven conversational agents, such as ChatGPT, in the healthcare sector. This article explores the consequences of overreliance on AI chatbots like ChatGPT in healthcare settings. It discusses the increasing use of AI chatbots for patient consultations, information dissemination, and decision support, highlighting their potential benefits in improving healthcare delivery efficiency and patient outcomes. The editorial explores the factors contributing to ChatGPT Dependency Disorder among healthcare professionals, such as convenience, lack of training, and time constraints, and examines the challenges and benefits associated with integrating AI chatbots in clinical workflows. It emphasizes the importance of maintaining a human-centered approach alongside AI technologies to optimize patient care outcomes and emphasizes the need for responsible integration of AI chatbots in healthcare settings to ensure ethical standards and patient safety. This article concludes by calling for further research and strategies to address ChatGPT Dependency Disorder and promote a balanced approach to leveraging AI technology in healthcare practice.

## Editorial

ChatGPT Dependency Disorder refers to the potential overreliance or attachment to AI-powered conversational agents like ChatGPT in healthcare practice. This phenomenon raises concerns about reduced human interaction, reliance on AI for decision-making, and potential psychological effects on healthcare professionals. Research has explored various aspects of AI chatbot usage in healthcare, such as its impact on clinical workflows, translation services, and the need for desensitization protocols for technology adoption. Understanding and addressing ChatGPT Dependency Disorder is crucial to maintaining a balance between AI assistance and human-centric care in healthcare settings.

AI chatbot is a computer program that uses AI to simulate conversations with users. The increasing use of AI chatbots like ChatGPT, IBM Watson Health's chatbots, Infermedica's Symptom Checker, Ada Health, Buoy Health, Your.MD, and Woebot in healthcare settings have been notable in recent years. These AI chatbots are being deployed for various purposes, such as patient consultations, symptom triaging, appointment scheduling, medication reminders, and providing health information. In inpatient consultations, AI chatbots like ChatGPT can interact with patients to gather initial information about symptoms, provide basic medical advice, and offer guidance on when to seek further medical attention [1]. For information dissemination, ChatGPT can be used to deliver personalized health education materials, answer frequently asked questions, and enhance health literacy among patients. Moreover, in decision support, ChatGPT can assist healthcare professionals by providing evidence-based recommendations, suggesting differential diagnoses, and aiding in clinical decision-making processes. These applications demonstrate the versatility and potential benefits of integrating ChatGPT in various aspects of healthcare delivery.

Healthcare institutions are leveraging AI chatbots to improve access to healthcare services, enhance patient engagement, streamline administrative tasks, and provide round-the-clock support to patients. Research studies have shown the potential of AI chatbots in improving healthcare delivery efficiency, patient outcomes, and overall healthcare experience [2,3]. As AI technologies continue to advance, the integration of AI chatbots like ChatGPT in healthcare settings is expected to grow further, revolutionizing the way healthcare services are delivered and accessed.

This article aims to shed light on the emerging issue of ChatGPT Dependency Disorder in healthcare practice and provoke further discussion on the responsible integration of AI chatbots in clinical settings.

ChatGPT dependency dynamics

The ease of obtaining instant information and support from ChatGPT may lead to professionals relying heavily on its quick responses, potentially diminishing their own critical thinking skills and medical knowledge. If healthcare professionals are not adequately trained on the limitations and proper use of AI chatbots like ChatGPT, they may become overly dependent on these tools without fully understanding when human intervention or verification is necessary. High workloads and time constraints in healthcare settings can drive professionals to seek quick solutions and shortcuts, making them more susceptible to developing a dependency on AI chatbots for decision-making. Understanding these factors can help healthcare institutions design appropriate training programs, guidelines, and support systems to mitigate ChatGPT Dependency Disorder among healthcare professionals.

Challenges and benefits associated with ChatGPT in clinical workflows

Ensuring the accuracy and reliability of information provided by AI chatbots is crucial for maintaining high-quality patient care and avoiding potential errors or misinterpretations. Safeguarding patient data and ensuring compliance with privacy regulations when using AI chatbots to handle sensitive health information is a significant challenge [4]. Healthcare professionals may face resistance or skepticism toward adopting AI chatbots in their workflows, leading to challenges in implementation and integration [5]. AI chatbots can streamline administrative tasks, improve appointment scheduling, and provide quick access to information, ultimately enhancing the efficiency of clinical workflows [2]. These chatbots utilize natural language processing and machine learning algorithms to interact with patients in a conversational manner, thereby decreasing the workload on administrative staff and enabling them to focus on more intricate tasks. Moreover, AI chatbots can send reminders, confirmations, and follow-ups, which ultimately reduces appointment no-shows and optimizes healthcare provider schedules. AI chatbots can engage patients through personalized interactions, health education, and continuous monitoring, leading to increased patient satisfaction and adherence to treatment plans. AI chatbots are utilized in the continuous monitoring of patients by providing real-time communication and support, collecting and analyzing patient data, and alerting healthcare providers to potential issues. These chatbots can interact with patients to gather information about their health status, symptoms, and medication adherence. By integrating with wearable devices and sensors, AI chatbots can continuously monitor vital signs, activity levels, and other health metrics. AI chatbots can assist healthcare professionals in decision-making processes by offering evidence-based recommendations, suggesting treatment options, and facilitating clinical documentation, thereby improving the quality of care provided. Directing these challenges while leveraging the benefits of AI chatbots in clinical workflows is essential for optimizing healthcare delivery and patient outcomes.

Ethical and psychological considerations

Ethical concerns surrounding the overreliance on AI chatbots for patient care include the risk of diminishing patient autonomy if healthcare decisions are solely based on AI chatbot recommendations, potentially reducing the patient's involvement in their own care and decision-making process. AI chatbots may perpetuate biases present in the training data, leading to discriminatory outcomes or unequal treatment of patients based on factors such as race, gender, or socioeconomic status. If errors or adverse events occur due to reliance on AI chatbots, determining accountability and responsibility can be challenging, raising questions about liability and the role of human oversight in healthcare decisions. Addressing these ethical concerns requires a balanced approach that integrates AI chatbots into patient care while upholding principles of patient autonomy, fairness, transparency, and accountability. Healthcare professionals and policymakers need to establish guidelines, protocols, and oversight mechanisms to ensure the ethical use of AI chatbots in patient care.

Dependency on ChatGPT among healthcare professionals may have various psychological impacts, including excessive reliance on ChatGPT for decision-making, which could lead to a decline in critical thinking skills among healthcare professionals, affecting their ability to assess complex medical situations independently. Relying heavily on ChatGPT may create a sense of pressure to always have immediate answers, potentially increasing stress and anxiety levels among healthcare professionals. Overdependence on AI chatbots like ChatGPT may erode the sense of professional autonomy and expertise among healthcare professionals, leading to a loss of professional identity and confidence in their own judgment. These potential psychological impacts underscore the importance of promoting a balanced approach to integrating AI technologies in healthcare practice, ensuring that healthcare professionals maintain their autonomy, critical thinking abilities, and overall well-being while utilizing tools like ChatGPT.

Implications of ChatGPT dependency in patient care

The frequent use of ChatGPT to interpret information or make diagnoses can result in errors due to its inability to fully comprehend context, its limited scope of training data, and the tendency of users to place undue faith in its responses without thoroughly evaluating them, potentially compromising the quality of patient care. Lack of human oversight and judgment in decision-making processes influenced by ChatGPT could result in suboptimal treatment plans or missed critical considerations. Healthcare professionals developing a dependency on ChatGPT may struggle with autonomous decision-making and critical thinking skills, affecting their ability to assess complex patient cases effectively. Overreliance on ChatGPT for clinical decision support may hinder the incorporation of clinical expertise, intuition, and patient context in decision-making processes, potentially leading to algorithm-driven or standardized care approaches [4]. Understanding the implications of ChatGPT Dependency Disorder on patient care and clinical decision-making underscores the importance of maintaining a balance between AI assistance and human judgment to ensure optimal healthcare outcomes.

Mitigation Strategies for AI Chatbot Overreliance in Healthcare

Continuous education and training: Provide healthcare professionals with ongoing education and training on the capabilities and limitations of AI chatbots. Foster critical thinking skills and reinforce the importance of human oversight in decision-making processes.

Human-AI collaboration: Encourage collaboration between healthcare professionals and AI chatbots to leverage the strengths of both parties. Emphasize the complementary role of AI chatbots in supporting rather than replacing human judgment in clinical decision-making.

Ethical guidelines and oversight: Establish clear ethical guidelines for the use of AI chatbots in patient care and decision-making. Implement oversight mechanisms to ensure transparency, accountability, and adherence to ethical standards in AI chatbot interactions. By implementing these strategies, healthcare institutions can promote responsible use of AI chatbots, mitigate the risks of excessive reliance, and maintain a balance between AI assistance and human-centric care in healthcare practice.

Future directions and recommendations

Conduct longitudinal studies to track the development and progression of ChatGPT Dependency Disorder among healthcare professionals over time. Explore how dependency levels change with prolonged use and the factors influencing this evolution. Utilize qualitative research methods, such as interviews and focus groups, to gain deeper insights into the psychological and behavioral aspects of ChatGPT Dependency Disorder. Explore the emotions, attitudes, and coping strategies of healthcare professionals dealing with dependency. Develop and evaluate intervention strategies aimed at mitigating ChatGPT Dependency Disorder. Test the effectiveness of training programs, guidelines, and support systems in promoting responsible AI chatbot usage and reducing dependency among healthcare professionals [1]. By pursuing these future research directions, we can enhance our understanding of ChatGPT Dependency Disorder, develop targeted interventions to address it, and promote a balanced approach to integrating AI technologies into healthcare practice.

Balanced Approach for AI Chatbots in Patient Care

Training and education: Provide comprehensive training programs for healthcare professionals on the capabilities and limitations of AI chatbots. Emphasize the importance of maintaining human oversight, critical thinking skills, and clinical judgment in conjunction with AI chatbot assistance [5].

Guidelines and protocols: Establish clear guidelines and protocols for the appropriate use of AI chatbots in patient care. Define the roles and responsibilities of healthcare professionals when interacting with AI chatbots and outline scenarios where human intervention is necessary.

Monitoring and evaluation: Implement mechanisms to monitor the impact of AI chatbots on patient care outcomes and healthcare workflows. Regularly evaluate the effectiveness of AI chatbot integration, solicit feedback from healthcare professionals, and make adjustments to optimize usage [3].

By implementing these recommendations, healthcare institutions can ensure that AI chatbots are integrated responsibly into patient care processes, maintaining a balance between leveraging AI technology and upholding the core values of human-centric healthcare delivery.

Conclusion

In conclusion, the discussion on ChatGPT Dependency Disorder in healthcare practice highlighted the potential psychological impacts on healthcare professionals, the impact on patient care quality and clinical decision-making, and strategies to mitigate risks associated with excessive reliance on AI chatbots. Factors contributing to ChatGPT Dependency Disorder include convenience, lack of training, and time constraints, while ethical concerns revolve around patient autonomy, bias, and accountability. To address ChatGPT Dependency Disorder, it is essential for healthcare institutions to provide education, establish guidelines, promote human-AI collaboration, and ensure ethical oversight. Future research directions should focus on longitudinal studies, qualitative research, and intervention strategies to better understand and address this disorder. Emphasizing a human-centered approach alongside AI technologies in healthcare delivery is crucial. Healthcare professionals should maintain their critical thinking skills, autonomy, and clinical judgment while leveraging AI chatbots as supportive tools. Balancing the benefits of AI technology with human expertise is essential to ensure optimal patient care outcomes and uphold ethical standards in healthcare practice.
